# Age, gender, and current living status were associated with perceived access to treatment among Canadians using a cross sectional survey

**DOI:** 10.1186/s12913-018-3215-6

**Published:** 2018-06-19

**Authors:** Catherine W. T. Chan, Amédé Gogovor, Marie-France Valois, Sara Ahmed

**Affiliations:** 1 Department of Epidemiology, Biostatistics and Occupational Health, McGill University, 1020 Pine Avenue West, Montreal, QC H3A 1A2 Canada; 2 Department of Medicine, McGill University, 687 Pine Avenue West, Ross Building, Montreal, QC H3A 1A1 Canada; 3 Centre for Outcomes Research and Evaluation, McGill University Health Centre, 5252 Boul. De Maisonneuve, Montreal, QC H4A 3S5 Canada; 4 School of Physical and Occupational Therapy, McGill University, 3654 Prom Sir-William-Osler, Montreal, QC H3G 1Y5 Canada; 5 Centre de recherche interdisciplinaire en réadaptation (CRIR), Constance Lethbridge Rehabilitation Center du CIUSSS de Centre-Ouest-de-l’Île-de-Montréal, 7005 de Maisonneuve Boulevard West, Montreal, QC H4B 1T3 Canada

**Keywords:** Chronic health conditions, Access to treatment, Health Care in Canada Survey

## Abstract

**Background:**

Access, particularly timely access, to care is the Canadian public’s most important healthcare concern. The drivers of perceived appropriateness of access to care among patients with at least one chronic health condition (CHC) are not, however, well defined. This study evaluated whether personal characteristics, self-reported health status and care received were associated with patients’ perception of effective access in managing a chronic illness.

**Methods:**

The study population (*n* = 619) was drawn from a representative sample of the adult Canadian population who reported having ≥1 CHC in the 2013–2014 Health Care in Canada survey. Ordinal regression, with the continuation ratio model, was used to evaluate association of perceived level of access to treatment with socio-demographic factors, perceived health status and care utilization experience.

**Results:**

Factors most closely associated with patients’ satisfaction with care access were: age, sex, current cohabitation, care affordability, and availability of support and information to help manage their CHCs. Individuals, particularly females, < 35 years, currently living alone, with poor access to professional support or information and who feel affordability of care has worsened over the past five years were more likely to report a poorer level of treatment access.

**Conclusions:**

Individuals living alone, who are younger, and women may be especially susceptible to lower perceived access to care of CHCs and a sense of pessimism about things not getting better. Further evaluation of the reasons behind these findings may help develop effective strategies to assist these populations to access the care they need.

**Electronic supplementary material:**

The online version of this article (10.1186/s12913-018-3215-6) contains supplementary material, which is available to authorized users.

## Background

For many Canadians, the universal, publicly-funded healthcare system plays an essential, if less than perfect, role in defining the nation’s identity [1]. All provincial and territorial health care insurance plans ensure patients have access to medically necessary services without having to pay at the point of care. The definition of medically necessary, however, can vary by jurisdiction [2, 3]. A study conducted by the Organization for Economic Co-operation and Development (OECD) noted marked variation in hospital admissions, and surgical and diagnostic procedures both within and between provinces and territories, beyond or below levels explained by patient need, which suggest the delivery of services without evidence of increased benefit to patients or the subsistence of unmet healthcare needs, respectively [3]. In the case that needs are unmet, it would be even more critical among those with chronic health conditions (CHCs). Responsible for 38 million deaths in 2012 (68% of all deaths), CHCs are a leading cause of death worldwide; and this statistic is projected to increase to 52 million by 2030 [4, 5]. It is a particularly grim statistic for patients with limited access to healthcare services needed to manage chronic conditions.

Although healthcare in Canada is universal, expenditures per capita are unevenly distributed among Canadians [6]. Approximately two-thirds of healthcare costs are spent on treating adults with multiple CHCs as these individuals tend to utilize healthcare services more often [4, 6]. Multiple CHCs also tend to manifest in individuals as they age [4]. More effective treatments for cancer, diabetes, chronic respiratory diseases, and other CHCs has contributed to greater life expectancy among patients and a steady rise in the prevalence of individuals with multiple chronic diseases [6]. As patients with greater demands on healthcare expenditures and resources continue to grow, the risk of unmet healthcare needs will steadily increase and the effective management of these cases will become extremely important.

It has been estimated that 1 in 7 adults with a chronic health condition (CHC) have unmet care needs, including inadequate access to care [7, 8]. Access is a complex, multidimensional concept that is central to charting the performance of healthcare systems [9]. Recent data from the 2013–2014 Health Care in Canada (HCIC) survey indicate, in fact, that lack of timely access is the most important care issue among the public [10]. An understanding of the population experiencing unmet healthcare needs will help to target healthcare services to the most vulnerable groups. In previous studies looking at the Canadian population, healthcare utilization (i.e., the number of consultations with a healthcare professional in the previous year), self-reported health status, the number of CHCs, the type of CHCs, age, sex, race, education, and income of the patient were shown to be associated with how well a patient perceives their healthcare needs to be met [8, 11]. These studies also reported that, compared to patients without a chronic condition, those with CHCs were more likely to report an unmet healthcare need [8, 11]; Ronksley et al. (2012) showed that the likelihood of this also increased with the number of CHCs [8]. Although the drivers of suboptimal access remain incompletely defined among patients with chronic conditions, public perception of access is practically important; it is negatively associated with rates of hospitalization for CHCs [12].

The objective of this study was to evaluate whether patient demography, perceived health status, number and severity of CHCs and care utilization experiences might drive perceptions of timeliness and effectiveness of care access among patients with CHCs. Using data from the 2013–2014 HCIC survey [10], we evaluated the relationship between such sociodemographic and health status/care variables and CHC patients’ perceived access to effective care.

## Methods

### Study population

The dataset consisted of responses from the online HCIC survey conducted by Pollara Inc. in 2013–2014, and, included representative samples of the Canadian adult public, health professionals (nurses, pharmacists, physicians) and administrators [13]. For the study analysis, only the public survey was utilized.

Canadians throughout all provinces were recruited, selecting those 18 years of age or older. An e-mail was sent to a random sample of eligible individuals. Those who accepted the e-mail invitation to participate in this study were provided with the survey. Through this process, the participants who responded to the survey were required to self-select into the sample at two stages: i.e., at the initial sign-up and then again by accepting the e-mail invitation if received.

No quotas were enforced during the sampling process. As such, weights were used for each set of responses accounted for age and sex distributions within province corresponding to the demographics in the 2011 Census to correct for the sampling method.

### Study variables

#### Primary outcome of interest

The outcome of interest was perceived access to treatment for the management of their CHC(s). In the survey, the participants were asked, “Do you have access to the treatments you need to manage your condition(s)?” Respondents were allowed to choose one of five possible responses: always, often, sometimes, rarely, or never. Due to the distribution of the responses in the dataset, cross-tabulations and regression analyses were performed grouping the latter three response options (i.e. sometimes, rarely, and never) into one category, while the respondents who answered “always” or “often” remained separate categories.

#### Independent variables

The Behavioural Model of Health Services Use described by Andersen [14] was used as a guide to determine the variables that would be of interest in the regression model. This model provides a framework to evaluate the factors that impact the use of health services and healthcare access [14]. Considering the factors raised in this framework, we incorporated variables that may be potential determinants of perceived access to treatment (refer to Tables 1 and 2, and Appendix 1 and Table A4.1 from Additional file 1).

### Statistical analysis

As our outcome of interest is ordinal and consists of three separate categories, the data was analyzed using an ordinal regression with the continuation ratio model to determine the association between the perceived access to treatment of the respondents and our predictors of interest [15]. Prior to univariate analyses, spearman correlations were performed in order to check if any independent variables were highly correlated with one another to ensure there would not be collinearity. If one or more variables were shown to be correlated (i.e., ρ ≥ 0.5), only one such variable was added into subsequent analysis.

Two separate ORs were produced for each model: OR_1_ compared the odds of the individuals in a specific category who feel they often/sometimes/rarely/never have access to treatment to the odds of those who always have access; OR_2_ compared the odds of those who feel they sometimes/rarely/never have access to those who often have access.

A multivariate ordinal regression analysis was performed to determine which factors might be potential drivers of suboptimal access to treatment. All statistical analysis was conducted using R 3.0.1 and SAS 9.3. Sensitivity analysis was conducted to ensure the robustness of the results.

## Results

### Descriptive statistics

Of the 1000 respondents, 619 individuals reported having at least one CHC. Among those who provided a response, 247 (40%) responded that they “always” had access to the treatment(s) required to manage their CHC, 170 (27%) responded that they “often” had access, while 202 (33%) claimed that they “sometimes/rarely/never” have access to treatment(s). Of the 619 respondents, the top three diagnoses reported were heart disease, stroke or high blood pressure (42%); arthritis (40%); and other unspecified health conditions (33%). More information regarding the diagnoses seen among the respondents can be found in Table A2.1 in Additional file 1. The distribution of sociodemographic characteristics and health-related measures among the respondents can be seen in Table 1.

**Table 1:** Characteristics of HCIC respondents from the public according to the perceived level of access to treatment(s) needed to manage chronic health condition(s) (CHC) (*n* = 619)

Characteristic	Total n (%)	Has access to treatment(s) needed to manage CHC(s):				
		Always	Often	Sometimes	Rarely	Never
		n (%)	n (%)	n (%)	n (%)	n (%)
		*n* = 247 (40)	*n* = 170 (27)	*n* = 131 (21)	*n* = 53 (9)	*n* = 18 (3)
Sex						
Female	320 (52)	116 (36)	83 (26)	84 (26)	29 (9)	8 (3)
Age						
< 35 years	70 (11)	10 (14)	27 (39)	26 (37)	6 (9)	1 (1)
35–44 years	80 (13)	32 (40)	22 (28)	13 (16)	9 (11)	4 (5)
45–54 years	137 (22)	42 (31)	38 (28)	38 (28)	15 (11)	4 (3)
55–64 years	162 (26)	61 (38)	47 (29)	33 (20)	16 (10)	5 (3)
≥65 years	170 (27)	102 (60)	36 (21)	21 (12)	7 (4)	4 (2)
Number of CHCs						
1	261 (42)	111 (43)	65 (25)	54 (21)	18 (7)	13 (5)
2	172 (28)	71 (41)	49 (28)	34 (20)	16 (9)	2 (1)
≥3	186 (30)	65 (35)	56 (30)	43 (23)	19 (10)	3 (2)
Household Income						
<$50,000	175 (28)	55 (31)	42 (24)	43 (25)	26 (15)	9 (5)
$50,000–$74,999	235 (38)	104 (44)	61 (26)	53 (23)	13 (6)	4 (2)
$75,000–$99,999	75 (12)	33 (44)	25 (33)	12 (16)	4 (5)	1 (1)
≥$100,000	64 (10)	26 (41)	21 (33)	10 (16)	5 (8)	2 (3)
Prefer not to say	70 (11)	29 (41)	21 (30)	13 (19)	5 (7)	2 (3)
Region^a^						
Ontario	237 (38)	106 (45)	66 (28)	46 (19)	11 (5)	8 (3)
Quebec	123 (20)	44 (36)	37 (30)	27 (22)	12 (10)	3 (2)
British Columbia	79 (13)	34 (43)	16 (20)	17 (22)	10 (13)	2 (3)
Alberta	61 (10)	20 (33)	20 (33)	17 (28)	4 (7)	0 (0)
Atlantic	80 (13)	32 (40)	20 (25)	15 (19)	9 (11)	4 (5)
Prairies	39 (6)	11 (28)	11 (28)	9 (23)	7 (18)	1 (3)
Health Insurance Plan						
Yes	312 (50)	136 (44)	90 (29)	61 (20)	21 (7)	4 (1)
No	307 (50)	111 (36)	80 (26)	70 (23)	32 (10)	14 (5)
Marital Status						
Single, never married	115 (19)	34 (30)	38 (33)	26 (23)	15 (13)	2 (2)
Married/Common-law	364 (59)	154 (42)	95 (26)	80 (22)	25 (7)	10 (3)
Divorced/separated	95 (15)	38 (40)	22 (23)	21 (22)	12 (13)	2 (2)
Widowed	41 (7)	20 (49)	13 (32)	3 (7)	1 (2)	4 (10)
Prefer not to say	4 (1)	1 (25)	2 (50)	1 (25)	0 (0)	0 (0)
Residence						
Urban	509 (82)	202 (40)	136 (27)	115 (23)	44 (9)	12 (2)
Rural	110 (18)	45 (41)	34 (31)	16 (15)	9 (8)	6 (5)
Currently live with another person						
Yes	445 (72)	189 (42)	118 (27)	94 (21)	33 (7)	11 (2)
No	174 (28)	58 (33)	52 (30)	37 (21)	20 (11)	7 (4)
Self-rated Health						
Excellent/Very Good	186 (30)	96 (52)	48 (26)	28 (15)	8 (4)	6 (3)
Good	246 (40)	102 (41)	71 (29)	47 (19)	20 (8)	6 (2)
Fair/Poor	187 (30)	49 (26)	51 (27)	56 (30)	25 (13)	6 (3)

^a^Territories were excluded since no individuals were sampled from this region

The list and frequency of the patient-reported reasons for poor access are shown in Table A5.1 in Additional file 1.

### Regression analysis

#### Personal characteristics

According to our fully adjusted model, age, gender, and current cohabitation with another individual were significantly associated with self-reported access to treatment among those with at least one CHC (Table 2).

**Table 2:** Adjusted odds ratios and associated 95% confidence intervals for the perceived likelihood of often/sometimes/rarely/never vs. always and sometimes/rarely/never vs. often receiving the access to treatment needed to manage CHCs

Variable of Interest	OR_1_^a^		OR_2_^b^		OR_cumulative_	
	OR	95% CI	OR	95% CI	OR	95% CI
Age						
< 35 years	1		1		1	
35 - 44 years	0.21	0.07, 0.66*	0.89	0.31, 2.54	0.48	0.24, 0.97*
45 - 54 years	0.30	0.10, 0.91*	1.24	0.49, 3.18	0.65	0.34, 1.26
55 - 64 years	0.31	0.10, 0.96*	1.84	0.69, 4.94	0.81	0.41, 1.61
> 65 years	0.13	0.04, 0.41*	1.24	0.40, 3.80	0.38	0.18, 0.78*
Sex						
Male	1		1		1	
Female	1.28	0.76, 2.17	2.09	1.15, 3.77*	1.50	1.04, 2.16*
Household income						
< $50,000	1		1		1	
$50,000 to $74,999	0.75	0.38, 1.49	0.71	0.33, 1.52	0.75	0.47, 1.21
$75,000 to $99,999	0.89	0.36, 2.22	0.47	0.17, 1.31	0.72	0.38, 1.37
> $100,000	1.05	0.38, 2.90	0.37	0.12, 1.09	0.68	0.34, 1.38
Prefer not to say	1.27	0.51, 3.18	0.56	0.21, 1.51	0.92	0.49, 1.73
Marital status						
Single	1		1		1	
Common-law/Married	0.92	0.38, 2.23	1.37	0.52, 3.60	1.05	0.57, 1.94
Divorced/Separated/Wi-dowed/Prefer not to say	0.49	0.21, 1.14	1.36	0.58, 3.19	0.74	0.42, 1.29
Residence						
Urban	1		1		1	
Rural	1.07	0.56, 2.04	0.58	0.28, 1.22	0.85	0.53, 1.35
Currently live with another person						
Yes	1		1		1	
No	2.40	1.04, 5.53*	1.43	0.56, 3.67	1.80	1.00, 3.25
Private Insurance						
Yes	1		1		1	
No	1.08	0.63, 1.86	1.08	0.59, 1.97	1.09	0.74, 1.58
Number of different types of prescription medications currently taking						
< 1	1		1		1	
2	1.28	0.50, 3.28	1.04	0.36, 3.02	1.09	0.57, 2.12
3	1.34	0.50, 3.58	1.20	0.38, 3.76	1.20	0.60, 2.41
> 4	1.48	0.61, 3.59	0.52	0.19, 1.41	1.02	0.55, 1.91
Self-rated Health						
Good	1		1		1	
Excellent	0.85	0.21, 3.50	0.34	0.06, 1.91	0.69	0.24, 1.99
Very Good	0.89	0.48, 1.66	1.12	0.54, 2.33	1.03	0.66, 1.61
Fair	1.07	0.55, 2.07	1.76	0.84, 3.70	1.45	0.91, 2.31
Poor	1.31	0.41, 4.17	1.33	0.48, 3.74	1.17	0.57, 2.40
Number of CHCs^c^						
1	1		1		1	
2	1.52	0.79, 2.91	1.27	0.61, 2.63	1.33	0.84, 2.10
≥3	2.03	0.90, 4.57	1.30	0.53, 3.20	1.50	0.86, 2.60
Type of Chronic Health Condition (CHC)						
Diagnosed with Diabetes						
No	1		1		1	
Yes	0.79	0.41, 1.53	0.60	0.26, 1.36	0.76	0.47, 1.22
Diagnosed with Heart Disease, Stroke, or High Blood Pressure						
No	1		1		1	
Yes	0.76	0.41, 1.38	0.91	0.46, 1.80	0.85	0.56, 1.29
Diagnosed with Cancer						
No	1		1		1	
Yes	0.73	0.32, 1.68	0.67	0.25, 1.79	0.70	0.39, 1.26
Perceived Affordability						
Summary of perceived affordability						
Did not perceive affordability as having worsened	1		1		1	
Worsened somewhat	0.98	0.47, 2.04	1.07	0.43, 2.65	1.02	0.59, 1.75
Worsened moderately	2.17	1.03, 4.58*	1.93	0.79, 4.68	1.92	1.13, 3.25*
Worsened a lot	2.35	0.99, 5.56	2.22	0.84, 5.85	2.25	1.22, 4.15*
Worsened completely	1.92	0.83, 4.39	1.52	0.62, 3.74	1.82	1.02, 3.26*
Type of Care Received						
Work with doctor or team of HCPs to manage CHC(s)						
Doctor	1		1		1	
Team	1.21	0.62, 2.37	0.58	0.26, 1.29	0.89	0.55, 1.44
Neither	1.85	0.73, 4.74	1.36	0.57, 3.24	1.82	0.99, 3.37
Receive the support needed from HCPs to manage CHC(s)						
Always	1		1		1	
Often	3.35	1.81, 6.19*	1.43	0.53, 3.82	2.86	1.75, 4.68*
Sometimes/Rarely/Never	5.01	2.37, 10.60*	3.75	1.31, 10.72*	5.47	3.09, 9.70*
Have access to the information needed to manage CHC(s)						
Always	1		1		1	
Often	6.43	3.59, 11.53*	0.56	0.24, 1.34	3.21	2.02, 5.10*
Sometimes/Rarely/Never	19.27	8.35, 44.45*	4.40	1.78,10.85*	14.71	8.25, 26.24*
Emergency Room (ER) Visits						
ER visit due to CHC(s)						
Never	1		1		1	
≤12 months	1.67	0.63, 4.40	2.13	0.74, 6.16	2.19	1.09, 4.41*
>12 months	1.31	0.77, 2.22	1.05	0.58, 1.91	1.17	0.81, 1.70
Adherence Variable						
Adherence to medications						
High	1		1		1	
Medium	1.78	0.97, 3.26	0.91	0.45, 1.84	1.36	0.88, 2.09
Low	1.49	0.59, 3.75	1.72	0.69, 4.29	1.51	0.83, 2.76
No prescription medications taken on a regular basis/Missing	2.00	0.76, 5.23	1.04	0.37, 2.97	1.48	0.75, 2.90

^*^Significant at *p* < 0.05

^a^OR_1_ represent odds ratio comparing the Often/Sometimes/Rarely/Never has access versus the Always has access

^b^OR_2_ represent odds ratio comparing the Sometimes/Rarely/Never has access versus the Often has access

^c^The variable with information about the number of CHCs was included despite showing non-significant values in a few initial models to ensure comparability between our analysis and that of a previous study [8]

Individuals over the age of 35 were less likely to report often, sometimes, rarely, or never having access to treatment compared to always having access to treatment. Furthermore, compared to those < 35 years, seniors (aged 65+) were 87% less likely to report their access to treatment as anything less than “Always” (OR_1_ = 0.13, 95% CI: 0.04–0.41). Females were twice as likely to report a level of access below “Often” compared to males (OR_2_ = 2.09, 95% CI: 1.15–3.77). Also, those currently living alone were 2.40 times (95% CI: 1.04–5.53) more likely to report poorer access (i.e., less than “Always” having access) than those living with another individual. Other personal characteristics did not have a statistically significant association with perceived access to treatment.

#### Chronic disease diagnosis

The number and the type of CHCs (diabetes, heart disease/stroke/ hyper-tension, or cancer) were not statistically associated with the perceived accessibility to treatment.

#### Perceived affordability

Compared to individuals who did not perceive affordability of the health system or its services as having worsened over the past five years, respondents who felt that affordability had worsened moderately were 2.17 times (95% CI: 1.03–4.58) more likely to report often/sometimes/rarely/never (vs. always) having access to treatment. None of the remaining associations were statistically significant.

#### Type of care received

Receiving support from health professionals and the information needed to manage CHCs were factors that were strongly associated with an individual’s perceived accessibility. Individuals who reported sometimes, rarely, or never receiving support from their HCPs were 5.01 times (95% CI: 2.37–10.60) more likely to report often to never (vs. always) receiving access to treatment and 3.75 times (95% CI: 1.31–10.72) more likely to report sometimes to never (vs. often) having access. Those who reported only often receiving support were also 3.35 times (95% CI: 1.81–6.19) more likely to perceive treatment access at a level below “always.” Similarly, individuals who felt that the information to manage their CHCs was sometimes, rarely, or never available were 19.27 times (95% CI: 8.35–44.45) more likely to report often to never (vs. always) having access to treatment, and 4.40 times (95% CI: 1.78–10.85) more likely to perceive treatment as sometimes to never (vs. often) accessible. Although not statistically significant, those without a doctor or HCP to help manage their condition were less likely to always or often feel that treatment was accessible.

#### Emergency department visits

The time since the last emergency department visit for a CHC-related issue was not a significant predictor of respondent perceptions on treatment access. The initial statistically significant effect observed in the univariate model (Table A4.1 in Additional file 1) was largely removed after adjusting for the personal characteristics of those surveyed.

#### Adherence to medication

The level of adherence to medications reported by the respondents initially conveyed a dose-response relationship after adjusting for the personal characteristics of the individual, where those who were less adherent were progressively more likely to report poorer access to treatment (Table A4.1 in Additional file 1). However, this factor was no longer a significant predictor of individuals’ perceptions of access after adjusting for other variables.

## Discussion

This study provided several insights into factors that are associated with perceived access to care. Our analysis supported that individuals with at least one CHC were more likely to report poorer access to treatment if they were <35 years, female, living alone, reported a more pessimistic view of care affordability, and self-reported less than always or often receiving adequate professional support and information to manage their conditions. In contrast to previous studies [11, 16, 17], income, residence (urban/rural), and self-rated health were not associated with self-reported access among those with CHCs. Additionally, the number and the type of CHCs were not significant predictors of access to care [8]. This contrast may be explained by the fact that, unlike prior studies, our sample included only individuals with ≥1 CHC.

A previous study using 2000/2001 Canadian Community Health Survey (CCHS) data reported that those who were female, younger, white, more highly educated, and earning a lower income were more likely to report suboptimal access to health care [11]. Although the HCIC survey did not ask for the participant’s level of education and ethnicity or cultural background, our results similarly showed that younger individuals and females were more likely to report poorer access to treatment. In Canada, a higher prevalence of chronic pain and depression is consistently reported among women [18, 19, 20, 21] – a statistic reflected in our sample where a slightly higher proportion of women (52%) reported having a CHC, and ~15% of females, as opposed to ~10% of males, reported being diagnosed with a mental health condition. The impact of these seemingly non-fatal chronic health disorders are often overlooked in terms of health service use despite effective treatment strategies in primary care [20, 22, 23]. As such, it is not surprising that women tend to perceive treatment access in a poorer light.

The perceptions of how affordable treatments or healthcare services have been shown to directly impact treatment access among Canadians. Although healthcare in Canada is publicly funded, out-of-pocket expenditures still occur. Results from the 2013 Commonwealth Fund International Health Policy Survey showed that 9 to 20% of Canadians spent over $1000 out-of-pocket on healthcare in that year [24]. In 2013, approximately 1 in 12 Canadians reported not filling prescriptions or missing a dose as a result of cost while 6% cited the cost of medical tests or treatments as the reason for not undergoing doctor-recommended procedures [24].

Our analysis showed that those currently living alone were more likely to report poor access to treatment. Using CCHS data, living arrangement was found to be significantly associated with unmet health care needs among young adults (aged 18–30 years), with those living alone being more likely to report unmet needs compared to those living with others, parents, or parents and spouse [25]. Another study reported that elderly individuals living only with their spouses were significantly more likely to receive preventive care (e.g. routine physical check-ups) [26]. Thus, living with another individual appears to increase healthcare encounters, which may in turn impact public perceptions of access to care.

In our model, it was observed that support from HCPs and the provision of information required to manage CHCs were associated with respondent perceptions of treatment access. Although the low frequencies for certain response categories may have resulted in less precise estimates of association, previous work has suggested that useful health information has the potential to facilitate a patient’s awareness of their disease, and they are better equipped to choose their care plan and carry it out [27]. Past studies have shown that patients with a deeper understanding of their illness and with support from others are able to better cope with their CHC through more effective use of available resources and engagement in behaviours that may influence the nature of their patient experience [27, 28]. Studies have also reported that individuals with chronic illness were more likely to use health information and self-care resources (e.g. self-care books, telemedicine services, or the Internet) [29, 30]. However, despite the various avenues for information available, and the empowering effect that this information provides, a 2005 survey found that patients still view physicians as the most trusted source for health information [27, 31]. As such, it is not surprising that our analysis showed an association between the availability of support from HCPs and respondent perceptions of treatment accessibility.

### Limitations

Gaps in chronic disease care has been reported between races in terms of the level of access and the availability of a usual source of medical care, while the level of education attained has also been shown to be associated with the likelihood of having access to treatments and care-seeking behaviours [11, 32, 33]. Due to limitations in our data, we were unable to adjust for these factors. We were also unable to adjust for whether or not the respondent received specialty care to assess its impact on access to care, particularly among those with more CHCs. As such, residual confounding may remain and should be investigated in future studies. Furthermore, the online administration of the HCIC could have hindered the participation of respondents with visual or physical impairments – patients who may not be able to respond to the questions without the aid of a caregiver. In the cases where these impairments are chronic, we may introduce selection bias into our study as data collection for respondents with these CHCs would be impeded. Lastly, some variables had small frequencies for some response categories (e.g. access to needed information), resulting in less precise estimates of association (Refer to Table A3.1 in Additional file 1).

### Strengths

Our study provided an overview of potential factors that may affect optimal access to treatment for patients with CHCs to manage their conditions. These results may provide a means to more effectively judge areas that healthcare programs or interventions should target to ensure improvements in chronic disease care. The online format of the survey also served as a strength since it may have encouraged more accurate reporting by respondents for sensitive information, and allowed for efficiency in survey distribution, data collection, and analysis (e.g. circumventing potential data entry errors). Although limitations in the data available through the HCIC survey precludes the making of causal inferences from the statistical analysis, our results will serve as a platform for future studies and provide insight on where resources should be allocated to ensure individuals with CHCs receive high quality care.

## Conclusions

Access to treatment was rated more poorly among those who are < 35 years, female, living alone, feel that the affordability of health services has worsened, and feel ill-equipped to manage their CHCs – making them more vulnerable to the consequences of untreated health conditions and its related comorbidities. Further evaluation of the reasons behind these findings can help develop effective strategies to help these populations access the care they need. Strategies to enable healthcare-seeking behaviours, as well as creating opportunities for social support have been shown to protect against unmet needs, and thus, have the potential to facilitate treatment access [25, 26]. Ultimately, however, these strategies may only improve perceptions of treatment access without effecting clinical outcomes [34, 35]. As such, evidence to show whether improved perceptions of treatment access can accurately reflect access levels and positively impact clinical outcomes remains a priority.

## Additional file

Additional file 1: This file provides additional data tables describing our study population and analysis. Appendix 1 provides a brief overview of how we constructed the summary variables for several multi-question concepts, including perceived affordability of the healthcare system or its services and medication adherence. Appendix 2 provides a frequency distribution of the CHCs reported by patients in the study population. Appendix 3 provides a frequency distribution for the additional covariates included in the ordinal regression model, outside of the sociodemographic factors seen in Table 1. These frequencies are stratified by the respondents’ reported level of access to treatment(s) needed to manage their CHCs. Appendix 4 provides the results from the univariate regression analysis. Appendix 5 looks into the self-reported reasons for not always having access to treatment to manage CHCs, cited by patients in the study population who perceive their level of access as “Often” or “Sometimes/Rarely/Never.”. (DOCX 84 kb)

## Abbreviations

CCHS: Canadian Community Health Survey
CHC: Chronic health condition
ER: Emergency room
HCIC: Health Care in Canada
HCP: Health care provider
