# Supplementary material for: Age, gender, and current living status were associated with perceived access to treatment among Canadians using a cross sectional survey
Source: BMC Health Serv Res. 2018 Jun 19;18:471. doi: 10.1186/s12913-018-3215-6 (PMC6006735; doi:10.1186/s12913-018-3215-6)
Supplement: Supplementary file 1 — This file provides additional data tables describing our study population and analysis. Appendix 1 provides a brief overview of how we constructed the summary variables for several multi-question concepts, including perceived affordability of the healthcare system or its services and medication adherence. Appendix 2 provides a frequency distribution of the CHCs reported by patients in the study population. Appendix 3 provides a frequency distribution for the additional covariates included in the ordinal regression model, outside of the sociodemographic factors seen in Table 1. These frequencies are stratified by the respondents’ reported level of access to treatment(s) needed to manage their CHCs. Appendix 4 provides the results from the univariate regression analysis. Appendix 5 looks into the self-reported reasons for not always having access to treatment to manage CHCs, cited by patients in the study population who perceive their level of access as “Often” or “Sometimes/Rarely/Never.”. (DOCX 84 kb) [file 12913_2018_3215_MOESM1_ESM.docx]

**Additional file 1**

**APPENDIX 1**

***Constructing Summary Variables for Affordability and Medication Adherence***

As the perceived affordability of the healthcare system or its services and adherence to medications were assessed through multi-part questions, summary variables were created for subsequent analyses. The perceived affordability of healthcare and its services was assessed through an 11-part question (Q. E2, parts a-k) in the survey. Respondents were placed into different categories of the summary variable depending on the number of times they responded that the healthcare system or the service had “worsened” in affordability, with a higher number of responses corresponding to poorer perception of affordability (0=affordability not perceived to be worsened; 1-3=worsened somewhat; 4-6=worsened moderately; 7-9=worsened a lot; 10-11=worsened completely).

Similarly, adherence to medication was assessed through a four-part question (Q. H3, parts a-d) in the survey. Responses of always, often, sometimes, or rarely to the statements asking whether respondents took their medications more frequently (part a) or less frequently (part b) than prescribed, or if these prescribed medications were taken at a higher dosage (part c) or lower dosage (part d) than instructed were counted to make the categories of the summary variable, with a fewer number of responses corresponding to better adherence (0=high adherence; 1-2=medium adherence; 3-4=low adherence).

**APPENDIX 2**

**Table A2.1** Frequency distribution of diagnoses for the respondents who reported having at least one CHC (n=619)

| **Diagnosis** | **Number of respondents*** | **Percentage out of the respondents with ≥1 CHC** |
| --- | --- | --- |
| ***Asthma, bronchitis or emphysema*** | 146 | 24% |
| ***Arthritis*** | 245 | 40% |
| ***Diabetes*** | 127 | 21% |
| ***Heart disease, stroke or high blood pressure*** | 258 | 42% |
| ***Osteoporosis*** | 57 | 9% |
| ***Cancer*** | 74 | 12% |
| ***A mental health condition*** | 156 | 25% |
| ***Any other health condition*** | 205 | 33% |
| **TOTAL RESPONDENTS** | **619** | **-** |

*Patients with more than one CHC will be counted in more than one diagnosis category

**APPENDIX 3**

**Table A3.1.** Frequency distribution for additional covariates included in the ordinal regression model (outside of the sociodemographic characteristics seen in Table 1), stratified by the perceived level of access to treatment(s) needed to manage chronic health condition(s) (CHC) (n=619)

| **Variable of Interest** | **Total** | | **Always has access** | | **Often has access** | | **Sometimes, Rarely, or Never has access** | |
| --- | --- | --- | --- | --- | --- | --- | --- | --- |
|  | **Freq.*** | **(% of n)** | **Freq.*** | **(% of Total)** | **Freq.*** | **(% of Total)** | **Freq.*** | **(% of Total)** |
|  | | | | | | | | |
| ***Number of different types of prescription medications currently taking*** | | | | | | | | |
| < 1 | 75 | (12) | 28 | (37) | 21 | (28) | 26 | (35) |
| 2 | 90 | (15) | 36 | (40) | 24 | (27) | 30 | (33) |
| 3 | 80 | (13) | 39 | (49) | 17 | (21) | 24 | (30) |
| > 4 | 239 | (39) | 96 | (40) | 78 | (33) | 65 | (27) |
| Missing | 135 | (22) | 48 | (36) | 30 | (22) | 57 | (42) |
|  |  |  |  |  |  |  |  |  |
| **Type of Chronic Health Condition (CHC)** | | | | | | | | |
|  |  |  |  |  |  |  |  |  |
| ***Diagnosed with Diabetes*** |  |  |  |  |  |  |  |  |
| No | 492 | (79) | 186 | (38) | 131 | (27) | 175 | (36) |
| Yes | 127 | (21) | 61 | (48) | 39 | (31) | 27 | (21) |
|  |  |  |  |  |  |  |  |  |
| ***Diagnosed with Heart Disease, Stroke, or High Blood Pressure*** | | | | | | | | |
| No | 361 | (58) | 124 | (34) | 99 | (27) | 138 | (38) |
| Yes | 258 | (42) | 123 | (48) | 71 | (28) | 64 | (25) |
|  |  |  |  |  |  |  |  |  |
| ***Diagnosed with Cancer*** |  |  |  |  |  |  |  |  |
| No | 545 | (88) | 207 | (38) | 151 | (28) | 187 | (34) |
| Yes | 74 | (12) | 40 | (54) | 19 | (26) | 15 | (20) |
|  |  |  |  |  |  |  |  |  |
| **Perceived Affordability** |  |  |  |  |  |  |  |  |
|  | | | | | | | | |
| ***Summary of perceived affordability*** | | | | | | | | |
| Did not perceive affordability as having worsened | 137 | (22) | 66 | (48) | 37 | (27) | 34 | (25) |
| Worsened somewhat | 145 | (23) | 73 | (50) | 35 | (24) | 37 | (26) |
| Worsened moderately | 136 | (22) | 49 | (36) | 45 | (33) | 42 | (31) |
| Worsened a lot | 52 | (8) | 19 | (37) | 16 | (31) | 17 | (33) |
| Worsened completely | 149 | (24) | 40 | (27) | 37 | (25) | 72 | (48) |
|  | | | | | | | | |
| **Type of Care Received** |  |  | | | | | | |
|  | | |  | |  | |  |  |
| ***Work with doctor or team of HCPs to manage CHC(s)*** | | | | | | | | |
| Doctor | 435 | (70) | 191 | (44) | 117 | (27) | 127 | (29) |
| Team | 105 | (17) | 44 | (42) | 36 | (34) | 25 | (24) |
| Neither | 79 | (13) | 12 | (15) | 17 | (22) | 50 | (63) |
|  | | | | | | | | |

***Freq. = Frequency**

**Table A3.1 (continued)**

| **Variable of Interest** | **Total** | | **Always has access** | | **Often has access** | | **Sometimes, Rarely, or Never has access** | |
| --- | --- | --- | --- | --- | --- | --- | --- | --- |
|  | **Freq.*** | **(% of n)** | **Freq.*** | **(% of Total)** | **Freq.*** | **(% of Total)** | **Freq.*** | **(% of Total)** |
|  | | | | | | | | |
| ***Receive the support needed from HCPs to manage CHC(s)*** | | | | | | | | |
| Always | 223 | (36) | 177 | (79) | 36 | (16) | 10 | (4) |
| Often | 177 | (29) | 44 | (25) | 86 | (49) | 47 | (27) |
| Sometimes/Rarely/Never | 219 | (35) | 26 | (12) | 48 | (22) | 145 | (66) |
|  |  |  |  |  |  |  |  |  |
| ***Have access to the information needed to manage CHC(s)*** | | | | | | | | |
| Always | 235 | (38) | 184 | (78) | 34 | (14) | 17 | (7) |
| Often | 201 | (32) | 52 | (26) | 105 | (52) | 44 | (22) |
| Sometimes/Rarely/Never | 183 | (30) | 11 | (6) | 31 | (17) | 141 | (77) |
|  |  |  |  |  |  |  |  |  |
| **Emergency Room (ER) Visits** | | | | | | | | |
|  | | | | | | | | |
| ***ER visit due to CHC(s)*** | | | | | | | | |
| Never | 192 | (31) | 91 | (47) | 47 | (24) | 54 | (28) |
| ≤12 months | 113 | (18) | 30 | (27) | 38 | (34) | 45 | (40) |
| >12 months | 314 | (51) | 126 | (40) | 85 | (27) | 103 | (33) |
|  | | | | | | | | |
| **Adherence Variable** |  |  |  |  |  |  |  |  |
|  | | | | | | | | |
| ***Adherence to medications*** | | | | | | | | |
| High | 245 | (40) | 128 | (52) | 60 | (24) | 57 | (23) |
| Medium | 167 | (27) | 57 | (34) | 57 | (34) | 53 | (32) |
| Low | 72 | (12) | 14 | (19) | 23 | (32) | 35 | (49) |
| No prescription medications taken on a regular basis/Missing | 135 | (22) | 48 | (36) | 30 | (22) | 57 | (42) |
|  | | | | | | | | |

***Freq. = Frequency**

**APPENDIX 4**

***Univariate Regression Analysis***

**Table A4.1.** Unadjusted odds ratios and associated 95% confidence intervals for the perceived likelihood of often/sometimes/rarely/never vs. always and sometimes/rarely/never vs. often receiving the access to treatment needed to manage CHCs

| **Variable of Interest** |  | **OR_1_^a^** | | | | | | | | | |  | | **OR_2_^b^** | |  | | **OR_cumulative_** | |  |
| --- | --- | --- | --- | --- | --- | --- | --- | --- | --- | --- | --- | --- | --- | --- | --- | --- | --- | --- | --- | --- |
|  | **OR** | | **95% CI** | | | | | | | | | | **OR** | | **95% CI** | | **OR** | | **95% CI** | |
| ***Age*** |  | |  | | | | | | | | | |  | |  | |  | |  | |
| < 35 years | 1 | |  | | | | | | | | | | 1 | |  | | 1 | |  | |
| 35 - 44 years | 0.25 | | 0.11, 0.56***** | | | | | | | | | | 0.97 | | 0.45, 2.07 | | 0.53 | | 0.31, 0.89* | |
| 45 - 54 years | 0.38 | | 0.18, 0.81***** | | | | | | | | | | 1.23 | | 0.64, 2.36 | | 0.74 | | 0.46, 1.18 | |
| 55 - 64 years | 0.28 | | 0.13, 0.58***** | | | | | | | | | | 0.94 | | 0.50, 1.79 | | 0.55 | | 0.35, 0.87* | |
| > 65 years | 0.11 | | 0.05, 0.23***** | | | | | | | | | | 0.73 | | 0.36, 1.46 | | 0.27 | | 0.17, 0.43* | |
|  |  | |  | | | | | | | | | |  | |  | |  | |  | |
| ***Sex*** |  | |  | | | | | | | | | |  | |  | |  | |  | |
| Male | 1 | |  | | | | | | | | | | 1 | |  | | 1 | |  | |
| Female | 1.37 | | 0.99, 1.89 | | | | | | | | | | 1.57 | | 1.04, 2.36***** | | 1.44 | | 1.12, 1.86* | |
|  |  | |  | | | | | | | | | |  | |  | |  | |  | |
| ***Region*** | | | | | |  | |  | |  |  | | | | | |  | |  | |
| Ontario | 1 | |  | | | | | | | | | | 1 | |  | | 1 | |  | |
| Québec | 1.45 | | 0.93, 2.28 | | | | | | | | | | 1.15 | | 0.66, 2.02 | | 1.32 | | 0.93, 1.88 | |
| BC | 1.07 | | 0.64, 1.79 | | | | | | | | | | 1.84 | | 0.91, 3.71 | | 1.30 | | 0.86, 1.97 | |
| Alberta | 1.66 | | 0.92, 3.00 | | | | | | | | | | 1.07 | | 0.53, 2.15 | | 1.38 | | 0.88, 2.16 | |
| Atlantic | 1.21 | | 0.73, 2.03 | | | | | | | | | | 1.42 | | 0.73, 2.77 | | 1.29 | | 0.86, 1.94 | |
| Prairies | 2.06 | | 0.98, 4.33 | | | | | | | | | | 1.57 | | 0.68, 3.61 | | 1.83 | | 1.05, 3.17* | |
|  |  | |  | | | | | | | | | |  | |  | |  | |  | |
| ***Household income*** | | | | |  | |  | |  | | | | | |  | |  | |  | |
| < $50,000 | 1 | |  | | | | | | | | | | 1 | |  | | 1 | |  | |
| $50,000 to $74,999 | 0.58 | | 0.38, 0.87***** | | | | | | | | | | 0.62 | | 0.37, 1.03 | | 0.59 | | 0.43, 0.81* | |
| $75,000 to $99,999 | 0.58 | | 0.33, 1.02 | | | | | | | | | | 0.37 | | 0.18, 0.75***** | | 0.49 | | 0.32, 0.76* | |
| > $100,000 | 0.67 | | 0.37, 1.21 | | | | | | | | | | 0.44 | | 0.21, 0.92***** | | 0.57 | | 0.36, 0.90* | |
| Prefer not to say | 0.65 | | 0.37, 1.15 | | | | | | | | | | 0.51 | | 0.25, 1.05 | | 0.59 | | 0.38, 0.92* | |
|  |  | |  | | | | | | | | | |  | |  | |  | |  | |
| ***Marital status*** | | | | | |  | |  | |  |  | | | | | |  | |  | |
| Single | 1 | | |  | | | | | | | | | 1 | |  | | 1 | |  | |
| Common-law/Married | 0.57 | | | 0.37, 0.90***** | | | | | | | | | 1.07 | | 0.64, 1.79 | | 0.75 | | 0.54, 1.05 | |
| Divorced/Separated/Widowed/Prefer not to say | 0.58 | | | 0.34, 0.97***** | | | | | | | | | 1.05 | | 0.57, 1.95 | | 0.75 | | 0.50, 1.11 | |
|  |  | | |  | | | | | | | | |  | |  | |  | |  | |
| ***Residence*** |  | | |  | | | | | | | | |  | |  | |  | |  | |
| Urban | 1 | | |  | | | | | | | | | 1 | |  | | 1 | |  | |
| Rural | 0.95 | | | 0.63, 1.45 | | | | | | | | | 0.73 | | 0.42, 1.24 | | 0.86 | | 0.62, 1.19 | |
|  |  | | |  | | | | | | | | |  | |  | |  | |  | |
| ***Currently live with another person*** | | | |  | | | | | | | | |  | |  | |  | |  | |
| Yes | 1 | | |  | | | | | | | | | 1 | |  | | 1 | |  | |
| No | 1.48 | | | 1.02, 2.13***** | | | | | | | | | 1.05 | | 0.68, 1.64 | | 1.29 | | 0.97, 1.71 | |
|  |  | | |  | | | | | | | | |  | |  | |  | |  | |
| ***Private Insurance*** |  | | |  | | | | | | | | |  | |  | |  | |  | |
| Yes | 1 | | |  | | | | | | | | | 1 | |  | | 1 | |  | |
| No | 1.36 | | | 0.99, 1.89 | | | | | | | | | 1.52 | | 1.01, 2.29***** | | 1.42 | | 1.10, 1.83* | |
|  |  | | |  | | | | | | | | |  | |  | |  | |  | |

*** Significant at p <0.05**

**Table A4.1 (continued)**

| **Variable of Interest** |  | | **OR_1_^a^** | | |  | | **OR_2_^b^** | |  | | **OR_cumulative_** | |  |
| --- | --- | --- | --- | --- | --- | --- | --- | --- | --- | --- | --- | --- | --- | --- |
|  | **OR** | | | **95% CI** | | | **OR** | | **95% CI** | | **OR** | | **95% CI** | |
|  |  | | | |  | |  | |  | |  | |  | |
| ***Number of different types of prescription medications currently taking*** | | | | | | | | | | | | |  | |
| < 1 | 1 | | | |  | | 1 | |  | | 1 | |  | |
| 2 | 0.85 | | | | 0.51, 1.41 | | 0.77 | | 0.41, 1.46 | | 0.82 | | 0.55, 1.21 | |
| 3 | 0.60 | | | | 0.35, 1.00 | | 0.87 | | 0.43, 1.77 | | 0.67 | | 0.44, 1.02 | |
| > 4 | 0.85 | | | | 0.58, 1.24 | | 0.51 | | 0.32, 0.83***** | | 0.70 | | 0.52, 0.94* | |
|  |  | | | |  | |  | |  | |  | |  | |
| ***Number of CHCs*** |  | | | |  | |  | |  | |  | |  | |
| 1 | 1 | | | |  | | 1 | |  | | 1 | |  | |
| 2 | | 1.05 | | | 0.71, 1.56 | | 0.81 | | 0.49, 1.35 | | 0.96 | | 0.70, 1.30 | |
| > 3 | 1.38 | | | | 0.93, 2.03 | | 0.89 | | 0.55, 1.44 | | 1.16 | | 0.86, 1.57 | |
|  |  | | | |  | |  | |  | |  | |  | |
| ***Self-rated Health*** |  | | | |  | |  | |  | |  | |  | |
| Good | 1 | | | |  | | 1 | |  | | 1 | |  | |
| Very good/Excellent | 0.66 | | | | 0.45, 0.97***** | | 0.85 | | 0.50, 1.44 | | 0.72 | | 0.53, 0.98* | |
| Fair/Poor | 2.00 | | | | 1.32, 3.02***** | | 1.66 | | 1.03, 2.67***** | | 1.83 | | 1.34, 2.50* | |
|  |  | | | |  | |  | |  | |  | |  | |
| ***Work with care provider to manage condition*** | | | | | | | | | | |  | |  | |
| Doctor | 1 | | | |  | | 1 | |  | | 1 | |  | |
| Team | 1.09 | | | | 0.71, 1.67 | | 0.64 | | 0.36, 1.13 | | 0.89 | | 0.64, 1.26 | |
| Neither | 4.37 | | | | 2.30, 8.31***** | | 2.71 | | 1.48, 4.96***** | | 3.47 | | 2.25, 5.37* | |
|  |  | | | |  | |  | |  | |  | |  | |
| ***Receive support from health professionals to manage condition*** | | | | | | | | | | | | | | |
| Always | 1 | | | |  | | 1 | |  | | 1 | |  | |
| Often | 11.63 | | | | 7.27, 18.62***** | | 1.97 | | 0.90, 4.32 | | 7.69 | | 5.15, 11.48* | |
| Sometimes | 24.05 | | | | 13.56, 42.64***** | | 7.11 | | 3.22, 15.72***** | | 20.77 | | 13.18, 32.73* | |
| Rarely/Never | 43.61 | | | | 17.81, 106.78***** | | 37.20 | | 12.48, 110.89***** | | 71.63 | | 35.53, 144.39* | |
|  |  | | | |  | |  | |  | |  | |  | |
| ***Have access to information you need to manage condition*** | | | | | | | | | | | | | | |
| Always | 1 | | | |  | | 1 | |  | | 1 | |  | |
| Often | 10.34 | | | | 6.64, 16.09***** | | 0.84 | | 0.42, 1.66 | | 5.81 | | 3.99, 8.46* | |
| Sometimes | 48.91 | | | | 23.23, 102.98***** | | 6.13 | | 3.01, 12.52***** | | 31.46 | | 19.18, 51.59* | |
| Rarely/Never | 90.20 | | | | 21.22, 383.35***** | | 97.88 | | 12.44, 769.99***** | | 199.47 | | 59.47, 669.08* | |
|  |  | | | |  | |  | |  | |  | |  | |
| **Affordability Variables** |  | | | |  | |  | |  | |  | |  | |
|  |  | | | |  | |  | |  | |  | |  | |
| ***Affordability of the health care system in general*** | | | | | | | | | | |  | |  | |
| Remained the same | 1 | | | |  | | 1 | |  | | 1 | |  | |
| Improved | 0.52 | | | | 0.27, 1.03 | | 0.55 | | 0.19, 1.59 | | 0.54 | | 0.30, 0.95* | |
| Worsened | 1.35 | | | | 0.94, 1.93 | | 1.32 | | 0.84, 2.08 | | 1.33 | | 1.01, 1.77* | |
| Don't know | 1.12 | | | | 0.64, 1.96 | | 1.96 | | 0.94, 4.09 | | 1.39 | | 0.89, 2.16 | |
|  |  | | | |  | |  | |  | |  | |  | |
| ***Affordability of specialty care*** | | | | | | |  | |  | |  | |  | |
| Remained the same | 1 | | | |  | | 1 | |  | | 1 | |  | |
| Improved | 0.48 | | | | 0.23, 1.01 | | 0.52 | | 0.15, 1.82 | | 0.49 | | 0.26, 0.93* | |
| Worsened | 1.92 | | | | 1.29, 2.86***** | | 1.73 | | 1.05, 2.87***** | | 1.83 | | 1.34, 2.50* | |
| Don't know | 0.99 | | | | 0.63, 1.56 | | 1.38 | | 0.74, 2.56 | | 1.11 | | 0.77, 1.61 | |

*** Significant at p <0.05**

**Table A4.1 (continued)**

|  |  | OR_1_^a^ | | |  | | OR_2_^b^ | |  | | OR_cumulative_ | |  |  |
| --- | --- | --- | --- | --- | --- | --- | --- | --- | --- | --- | --- | --- | --- | --- |
|  | OR | | 95% CI | | | OR | | 95% CI | | OR | | 95% CI | | |
| **Affordability variables (cont’d)** | | |  | | |  | |  | |  | |  | | |
|  | | |  | | |  | |  | |  | |  | | |
| ***Affordability of new medicines*** | | | |  | |  | |  | |  | |  | | |
| Remained the same | 1 | | |  | | 1 | |  | | 1 | |  | | |
| Improved | 1.02 | | | 0.55, 1.89 | | 0.50 | | 0.21, 1.22 | | 0.80 | | 0.49, 1.33 | | |
| Worsened | 1.70 | | | 1.13, 2.57***** | | 1.41 | | 0.82, 2.43 | | 1.60 | | 1.15, 2.22* | | |
| Don't know | 1.33 | | | 0.80, 2.21 | | 1.23 | | 0.63, 2.42 | | 1.30 | | 0.87, 1.95 | | |
|  | | |  | | |  | |  | |  | |  | | |
| ***Affordability of home care*** | | | | | |  | |  | |  | |  | | |
| Remained the same | 1 | |  | | | 1 | |  | | 1 | |  | | |
| Improved | 0.76 | | 0.35, 1.62 | | | 0.80 | | 0.27, 2.39 | | 0.76 | | 0.41, 1.43 | | |
| Worsened | 1.69 | | 1.13, 2.52***** | | | 1.12 | | 0.67, 1.87 | | 1.44 | | 1.05, 1.97* | | |
| Don't know | 1.18 | | 0.75, 1.85 | | | 1.14 | | 0.63, 2.08 | | 1.17 | | 0.82, 1.68 | | |
|  |  | |  | | |  | |  | |  | |  | | |
| ***Affordability of long term care*** | | | | | |  | |  | |  | |  | | |
| Remained the same | 1 | |  | | | 1 | |  | | 1 | |  | | |
| Improved | 0.94 | | 0.42, 2.08 | | | 0.74 | | 0.25, 2.19 | | 0.86 | | 0.45, 1.63 | | |
| Worsened | 1.38 | | 0.91, 2.08 | | | 1.15 | | 0.67, 1.98 | | 1.29 | | 0.93, 1.79 | | |
| Don't know | 1.24 | | 0.77, 2.02 | | | 1.27 | | 0.67, 2.40 | | 1.26 | | 0.85, 1.85 | | |
|  |  | |  | | |  | |  | |  | |  | | |
| ***Affordability of non-urgent surgery*** | | | | | |  | |  | |  | |  | | |
| Remained the same | 1 | |  | | | 1 | |  | | 1 | |  | | |
| Improved | 0.81 | | 0.37, 1.75 | | | 0.30 | | 0.08, 1.11 | | 0.61 | | 0.32, 1.16 | | |
| Worsened | 2.04 | | 1.39, 3.00***** | | | 1.82 | | 1.13, 2.94***** | | 1.95 | | 1.45, 2.64* | | |
| Don't know | 1.19 | | 0.77, 1.82 | | | 1.26 | | 0.72, 2.22 | | 1.22 | | 0.86, 1.71 | | |
|  |  | | |  | |  | |  | |  | |  | | |
| ***Affordability of diagnostic procedures*** | | | | | |  | |  | |  | |  | | |
| Remained the same | 1 | |  | | | 1 | |  | | 1 | |  | | |
| Improved | 0.74 | | 0.40, 1.37 | | | 0.84 | | 0.33, 2.12 | | 0.77 | | 0.46, 1.29 | | |
| Worsened | 2.12 | | 1.45, 3.11***** | | | 1.71 | | 1.06, 2.76***** | | 1.93 | | 1.44, 2.60* | | |
| Don't know | 1.36 | | 0.85, 2.16 | | | 2.12 | | 1.14, 3.95***** | | 1.61 | | 1.11, 2.33* | | |
|  |  | |  | | |  | |  | |  | |  | | |
| ***Affordability of hospice palliative and end-of-life care*** | | | | | |  | |  | |  | |  | | |
| Remained the same | 1 | | |  | | 1 | |  | | 1 | |  | | |
| Improved | 0.57 | | | 0.30, 1.07 | | 0.85 | | 0.32, 2.25 | | 0.63 | | 0.37, 1.08 | | |
| Worsened | 2.23 | | | 1.43, 3.49***** | | 1.70 | | 0.98, 2.96 | | 1.98 | | 1.40, 2.79* | | |
| Don't know | 1.20 | | | 0.79, 1.83 | | 1.35 | | 0.77, 2.37 | | 1.26 | | 0.90, 1.76 | | |
|  |  | | |  | |  | |  | |  | |  | | |
| ***Affordability of mental health services*** | | | | | |  | |  | |  | |  | | |
| Remained the same | 1 | | |  | | 1 | |  | | 1 | |  | | |
| Improved | 0.75 | | | 0.37, 1.54 | | 0.79 | | 0.29, 2.13 | | 0.77 | | 0.43, 1.37 | | |
| Worsened | 1.50 | | | 0.99, 2.27 | | 1.55 | | 0.93, 2.56 | | 1.51 | | 1.10, 2.07* | | |
| Don't know | 0.82 | | | 0.53, 1.25 | | 1.37 | | 0.78, 2.41 | | 0.98 | | 0.70, 1.38 | | |
|  |  | | |  | |  | |  | |  | |  | | |

*** Significant at p <0.05**

**Table A4.1 (continued)**

|  |  | OR_1_^a^ | | |  | | OR_2_^b^ | |  | | OR_cumulative_ | |  |
| --- | --- | --- | --- | --- | --- | --- | --- | --- | --- | --- | --- | --- | --- |
|  | OR | | 95% CI | | | OR | | 95% CI | | OR | | 95% CI | |
| **Affordability Variables (cont’d)** | | |  | | |  | |  | |  | |  | |
|  | | |  | | |  | |  | |  | |  | |
| ***Affordability of treatments (e.g. rehabilitation, physical therapy, etc.)*** | | | | | | | | | |  | |  | |
| Remained the same | 1 | |  | | | 1 | |  | | 1 | |  | |
| Improved | 0.73 | | 0.37, 1.41 | | | 0.25 | | 0.08, 0.78* | | 0.54 | | 0.31, 0.94* | |
| Worsened | 1.75 | | 1.20, 2.56***** | | | 1.45 | | 0.89, 2.34 | | 1.63 | | 1.21, 2.19* | |
| Don't know | 1.02 | | 0.63, 1.65 | | | 1.22 | | 0.63, 2.34 | | 1.08 | | 0.73, 1.60 | |
|  |  | |  | | |  | |  | |  | |  | |
| ***Affordability of screening/preventative tests and programs*** | | | | | | | |  | |  | |  | |
| Remained the same | 1 | |  | | | 1 | |  | | 1 | |  | |
| Improved | 0.60 | | 0.33, 1.10 | | | 0.48 | | 0.18, 1.30 | | 0.57 | | 0.34, 0.96* | |
| Worsened | 1.80 | | 1.22, 2.65***** | | | 2.25 | | 1.38, 3.64***** | | 1.96 | | 1.45, 2.64* | |
| Don't know | 1.30 | | 0.82, 2.04 | | | 1.93 | | 1.07, 3.47***** | | 1.51 | | 1.05, 2.15* | |
|  |  | |  | | |  | |  | |  | |  | |
| **Type of Chronic Health Condition (CHC)** | | | | | |  | |  | |  | |  | |
|  | | | | | |  | |  | |  | |  | |
| ***Diagnosed with Asthma, bronchitis or emphysema*** | | | | | |  | |  | |  | |  | |
| No | 1 | |  | | | 1 | |  | | 1 | |  | |
| Yes | 1.32 | | 0.90, 1.94 | | | 1.73 | | 1.07, 2.80***** | | 1.47 | | 1.09, 1.99* | |
|  |  | |  | | |  | |  | |  | |  | |
| ***Diagnosed with Arthritis*** | | | | | |  | |  | |  | |  | |
| No | 1 | |  | | | 1 | |  | | 1 | |  | |
| Yes | 1.52 | | 1.09. 2.13***** | | | 1.20 | | 0.79. 1.80 | | 1.39 | | 1.07. 1.79* | |
|  |  | | |  | |  | |  | |  | |  | |
| ***Diagnosed with Diabetes*** | | | | | |  | |  | |  | |  | |
| No | 1 | |  | | | 1 | |  | | 1 | |  | |
| Yes | 0.66 | | 0.44, 0.97***** | | | 0.52 | | 0.30, 0.89***** | | 0.61 | | 0.44, 0.83* | |
|  |  | |  | | |  | |  | |  | |  | |
| ***Diagnosed with Heart disease, stroke or high blood pressure*** | | | | | | | |  | |  | |  | |
| No | 1 | |  | | | 1 | |  | | 1 | |  | |
| Yes | 0.57 | | 0.41, 0.80***** | | | 0.65 | | 0.42, 0.99***** | | 0.60 | | 0.46, 0.78* | |
|  |  | | |  | |  | |  | |  | |  | |
| ***Diagnosed with Osteoporosis*** | | | | | |  | |  | |  | |  | |
| No | 1 | | |  | | 1 | |  | | 1 | |  | |
| Yes | 0.72 | | | 0.41, 1.24 | | 1.76 | | 0.80, 3.87 | | 0.97 | | 0.62, 1.52 | |
|  |  | | |  | |  | |  | |  | |  | |
| ***Diagnosed with Cancer*** |  | | |  | |  | |  | |  | |  | |
| No | 1 | | |  | | 1 | |  | | 1 | |  | |
| Yes | 0.52 | | | 0.32, 0.85***** | | 0.64 | | 0.31, 1.30 | | 0.56 | | 0.37, 0.83* | |
|  |  | | |  | |  | |  | |  | |  | |

*** Significant at p <0.05**

**Table A4.1 (continued)**

|  |  | OR_1_^a^ | | |  | | OR_2_^b^ | |  | | OR_cumulative_ | |  |
| --- | --- | --- | --- | --- | --- | --- | --- | --- | --- | --- | --- | --- | --- |
|  | OR | | 95% CI | | | OR | | 95% CI | | OR | | 95% CI | |
|  |  | |  | | |  | |  | |  | |  | |
| ***Diagnosed with a mental health condition*** | | | | | |  | |  | |  | |  | |
| No | 1 | |  | | | 1 | |  | | 1 | |  | |
| Yes | 2.35 | | 1.57, 3.51***** | | | 1.11 | | 0.71, 1.72 | | 1.69 | | 1.26, 2.26* | |
|  |  | |  | | |  | |  | |  | |  | |
| ***Diagnosed with any other CHC*** | | | | | |  | |  | |  | |  | |
| No | 1 | |  | | | 1 | |  | | 1 | |  | |
| Yes | 2.06 | | 1.44, 2.95***** | | | 1.08 | | 0.71, 1.64 | | 1.58 | | 1.20, 2.06* | |
|  |  | | |  | |  | |  | |  | |  | |
| ***Hospitalization due to CHC*** | | | | | |  | |  | |  | |  | |
| Never | 1 | | |  | | 1 | |  | | 1 | |  | |
| < 1 month | 1.30 | | | 0.42, 3.99 | | 0.63 | | 0.16, 2.46 | | 0.97 | | 0.42, 2.28 | |
| > 1 to < 3 months | 1.03 | | | 0.38, 2.80 | | 0.79 | | 0.22, 2.85 | | 0.93 | | 0.42, 2.08 | |
| > 3 to < 6 months | 2.02 | | | 0.70, 5.78 | | 1.05 | | 0.35, 3.20 | | 1.51 | | 0.71, 3.22 | |
| > 6 to < 12 months | 1.62 | | | 0.78, 3.36 | | 0.46 | | 0.20, 1.09 | | 0.96 | | 0.57, 1.63 | |
| > 12 to < 24 months | 0.66 | | | 0.36, 1.24 | | 1.80 | | 0.70, 4.67 | | 0.90 | | 0.54, 1.50 | |
| > 24 months | 1.16 | | | 0.81, 1.67 | | 0.94 | | 0.59, 1.49 | | 1.07 | | 0.80, 1.43 | |
|  |  | | |  | |  | |  | |  | |  | |
| ***ER visit due to CHC*** |  | | |  | |  | |  | |  | |  | |
| Never | 1 | | |  | | 1 | |  | | 1 | |  | |
| < 1 month | 3.15 | | | 1.00, 9.93 | | 2.18 | | 0.64, 7.40 | | 2.75 | | 1.19, 6.33* | |
| > 1 to < 3 months | 5.40 | | | 1.81, 16.16***** | | 0.44 | | 0.17, 1.11 | | 1.47 | | 0.80, 2.70 | |
| > 3 to < 6 months | 1.60 | | | 0.68, 3.80 | | 1.45 | | 0.49, 4.29 | | 1.56 | | 0.80, 3.08 | |
| > 6 to < 12 months | 2.01 | | | 0.99, 4.10 | | 1.23 | | 0.53, 2.85 | | 1.67 | | 0.97, 2.86 | |
| > 12 to < 24 months | 1.14 | | | 0.64, 2.03 | | 2.42 | | 1.03, 5.69***** | | 1.47 | | 0.91, 2.36 | |
| > 24 months | 1.40 | | | 0.96, 2.05 | | 0.89 | | 0.54, 1.48 | | 1.19 | | 0.88, 1.61 | |
|  |  | | |  | |  | |  | |  | |  | |
| **Adherence Variables** |  | | |  | |  | |  | |  | |  | |
|  |  | | |  | |  | |  | |  | |  | |
| ***Adherence to medications*** |  | | |  | |  | |  | |  | |  | |
| High | 1 | | |  | | 1 | |  | | 1 | |  | |
| Medium | 2.11 | | | 1.41, 3.17***** | | 0.98 | | 0.58, 1.65 | | 1.59 | | 1.15, 2.18* | |
| Low | 4.53 | | | 2.40, 8.55***** | | 1.60 | | 0.85, 3.03 | | 2.84 | | 1.83, 4.39* | |
| No prescription medications taken on a regular basis/Missing | 1.98 | | | 1.29, 3.06* | | 2.00 | | 1.13, 3.54* | | 2.05 | | 1.45, 2.89* | |

*** Significant at p <0.05**

**^a^ OR_1_ represent odds ratio comparing the Often/Sometimes/Rarely/Never has access to the Always has access**

**^b^ OR_2_ represent odds ratio comparing the Sometimes/Rarely/Never has access to the Often has access**

**APPENDIX 5**

**Table A5.1.** Reported reasons for not always having access to treatment to manage CHCs by the respondents who perceive their level of access as “Often” or “Sometimes/Rarely/Never”

| Reason | Self-reported level of access to treatment: | |
| --- | --- | --- |
|  | **Often**  **n (%)**  **n=170** | **Sometimes/Rarely/Never**  **n (%)**  **n=202** |
| Can't afford the treatment* | 55 (32) | 91 (45) |
| Can't get to or otherwise access the treatment* | 39 (23) | 56 (28) |
| Did not get referred to treatment in time* | 57 (34) | 51 (25) |
| Other* | 31 (18) | 69 (34) |

*Respondents may report more than one reason and will be counted more than once across the reason categories.

Late referral to treatment was cited most frequently as the reason for poor access among those claiming to “often” have access (34%), whereas unaffordability was the frontrunner for 45% of respondents who reported only “sometimes/rarely/never” having access. Other reasons reported included long wait times, distance from treatment centres or lack of available treatment, and unreachability or lack of a regular doctor, with nine respondents stating that treatment for their CHC was unnecessary.
